# Food insecurity as a risk factor for obesity: A review

**DOI:** 10.3389/fnut.2022.1012734

**Published:** 2022-09-26

**Authors:** Diana Carvajal-Aldaz, Gabriela Cucalon, Carlos Ordonez

**Affiliations:** Facultad de Ciencias de la Vida, Escuela Superior Politécnica del Litoral (ESPOL), Guayaquil, Ecuador

**Keywords:** obesity, food insecurity, paradox, food access, overweight

## Abstract

Obesity is considered a 21^st^-century epidemic and it is a metabolic risk factor for Non-Communicable Diseases such as cardiovascular diseases, type 2 diabetes, metabolic syndrome, hypertension, some types of cancer, among others. Thus, its prevention and treatment are important public health concerns. Obesity within the context of food insecurity adds an additional layer of complexity to the current obesity epidemic. Efficient policies and interventions ought to take into consideration the effects of food insecurity on the risks of developing obesity among food insecure households. This review aims to analyze the recent available evidence around the obesity – food insecurity paradox. Most of the literature has consistently shown that there is a significant association between food insecurity and obesity, specifically in women of high-income countries. However, mechanisms explaining the paradox are still lacking. Even though researchers have tried to analyze the issue using different individual and societal variables, these studies have failed to explain the mediatory mechanisms of the food insecurity–obesity relationship since the proposed mechanisms usually lack strength or are purely theoretical. The research focus should shift from cross-sectional models to other research designs that allow the exploration of pathways and mechanisms underlying the food insecurity and obesity relationship, such as longitudinal studies, which will hopefully lead to consecutive research testing the effectiveness of different approaches and scale up such interventions into diverse contexts among those affected by obesity and the different degrees of food insecurity.

## Introduction

In recent years, food insecurity (FI) has been exacerbated because of the COVID-19 pandemic and the increase of conflicts between nations, which affect food affordability worldwide. FI not only refers to the lack of access to acquire food (1), but itincludes its nutritional quality and food safety. In 2021, it was estimated that about 41 percent of the world population suffered some degree of FI, ranging from moderate to severe (2). Poverty is a key factor that contributes to FI and directly triggers the consumption of unhealthy diets among the low- and middle-income populations. Poverty strikes mainly African, Latin American, and Caribbean populations (2). Moreover, the Middle East, North Africa and Latin America are the low- and middle-income regions with the highest rate of obesity (3). Consequently, FI and poverty not only contribute to the rise of undernutrition, but it can also increase the prevalence of obesity.

The understanding of the coexistence of both food insecurity and obesity, although it might seem contradictory, is relevant for generating further public health actions. Obesity is considered a 21st-century epidemic (4), thus it is an important public health concern (5). This multifactorial chronic disease is influenced by social and environmental factors and not only by an altered nutritional behavior or a genetic disorder (6). Obesity is a metabolic risk factor for Non-Communicable Diseases (NCD) like type 2 diabetes, cardiovascular diseases, metabolic syndrome, hypertension, some types of cancer, among others (7). The treatment of these NCD that are associated with obesity is expensive (8) and it is estimated that obese individuals have a 30 percent higher medical expenses compared to individuals with a normal weight (9). The obese population is concentrated mainly in low- and middle-income countries (10).

Obesity within the context of FI adds an additional layer of complexity to the current obesity epidemic. Efficient policies and interventions ought to take into consideration the effects of FI on the risks of developing obesity among food insecure households. Therefore, this review aims to analyze the recent available evidence around the obesity - FI paradox.

## Food insecurity

Several reasons have interfered with attaining world hunger Sustainable Development Goals (SDGs) and diminishing malnutrition in all its forms by 2030 (11). Moreover, the impact of the COVID-19 pandemic has emphasized the need for contemplating how to address the major influencers of the global FI and malnutrition situation and its consequences on the population's health (2).

Food security has been defined by the Rome Declaration on World Food Security 1996 as as a state in which “all individuals, always, have physical, social, and economic access to sufficient, safe, and nutritious food to meet their dietary needs and food preferences for an active and healthy life” (12) whereas FI is the state where there is limited, inadequate, or unreliable availability or access to obtain nutritionally sufficient and safe foods in socially acceptable ways (1, 13, 14). This may be due to financial or social reasons, poverty, urbanization, environmental changes, and agricultural policy (15). FI around the world shows persistent regional disparities, Africa being the most affected region with 20 percent of its population facing hunger in 2021 compared to 9.1 percent in Asia, 8.6 percent in Latin America and the Caribbean, 5.8 percent in Oceania, and < 2.5 percent in Northern America and Europe, respectively (2).

As for 2021, the prevalence of moderate FI has remained somewhat unaffected in comparison to 2020. However, the prevalence of severe FI increased, this suggests that those who were already facing moderate FI deteriorated their situation because of the COVID-19 pandemic (11). In 2021, around29.3 was moderately food insecure, and 11.7 percent was severely food insecure (2). FI has been linked to chronic diseases, such as obesity, diabetes, pulmonary diseases, cardiovascular diseases, resulting in a decrease in quality of life, mental health issues, and increased mortality rates (16, 17).

According to Food Insecurity Experience Scale (FIES), FI can be experienced at different levels of severity. Moderate food insecurity characterizes by reduced accessibility to either quality and/or quantity of food, due to lack of financial resources or other resources. It can increase the risk of stunting in children, micronutrient deficiencies, or obesity in adulthood (18). On the other hand, severe food insecurity is at the extreme of the scale, meaning that people who fall into this categoryhave no food consumption for at least a day or more. This group are those called the “hungry” (19). Approximately 702–828 million people in the world faced hunger in 2021 (20).

## Poverty

If there are not enough resources to cover the necessities of life such as food, clean water, shelter, clothing, healthcare, education, and even transportation, that could put well-being at risk (19). Specifically, it could lead to even more people finding healthy diets unaffordable. To define poverty, the World Bank and FAO use extreme poverty as a reference to measure the situation. Those living on less than USD 1.90 a day (2011 PPP prices) in a country each year are considered to be extremely poor (2, 21) while people living on between $1.90-$3.10 per day are moderately poor (2).

The COVID-19 pandemic and the war in Ukraine have influenced on food prices, food supply chains and are affecting the economic recovery among countries. Poverty has increased specifically in Sub-Saharan Africa, Latin America, and the Caribbean (2). The poorest countries are not the only ones being affected but also households with 60 percent lower global income distribution (2). Pre-COVID-19 calculations estimated that daily per capita household incomes would grow from $7.15 in 2019 to $7.44 in 2021 (22).

## Obesity

The rise in obesity and other forms of malnutrition could partly be a result of moderate FI. Highly energy-dense processed foods that are high in saturated fats, sugars, and sodium are consumed more often than micronutrient-dense quality foods (23). Energy-dense foods may help meet daily caloric requirements, but essential nutrients are missing. Therefore, in many countries, undernutrition and obesity coexist and both can be consequences of FI (24).

The relationship between obesity and FI has been studied since its prevalence has been increasing worldwide. Weight gain happens when individuals ingest more energy than they expend. Adipose tissue accumulation is an adaptive strategy used to defend themselves against periods when food is unavailable (16, 24). Consequently, the optimal level of body fat relies on access to food. Obesity is a chronic, relapsing, multifactorial disease, that starts early in life, and childhood obesity is now a growing public health concern where early prevention is crucial (10). From an epidemiological perspective, the most widely used method to identify obesity is body mass index. In adults, a BMI of ≥30 kg/m2 is used to define the prevalence of obesity (24).

By 2030, 1 in five women and one in seven men, will be obese. Nations will not only fail to achieve the 2025 WHO target to halt the rise in obesity at 2010 levels, but the number of individuals living with obesity is on its course to a 2-fold increase worldwide (10). The highest prevalence of obesity is found in low- and middle-income countries, with numbers steeply increasing compared to 2010 (10). A new measure called the Obesity-Non-Communicable Disease Preparedness Index indicates that all of the 30 most prepared countries are high-income countries, whereas the 30 least prepared countries are all low- and middle-income countries (10).

Therefore, additional studies are needed to better understand underlying mechanisms, associated risks, and effective strategies to mitigate these public health concerns.

## The obesity – food insecurity paradox

The coexistence of both obesity and FI has drawn the attention of researchers since it seems contradictory that people with limited access to food can become obese. In the last 5 years, a few articles have been published related to this topic.

One of the plausible mechanisms that could explain this paradox is that FI leads to a low dietary quality which emphasizes the consumption of energy dense foods. Kowaleski-Jones and colleagues aimed to explain the association between obesity and FI through potential mediating risk factors such as lack of access to healthy foods, physical activity, energy intake, stress, access to healthcare and marital status using the data obtained from the 2007–2008 National Health and Examination Surveys NHANES (25). Even though the aforementioned variables were significantly associated, none of them served as mediators of the relationship between FI and obesity after being tested using multiple regression techniques. The positive association between FI and elevated BMI was true only in women, this finding is consistent with other studies (16, 26–28).

On the other hand, Potochnick et al. explored the health implications that FI has among Hispanic/Latino youth living in the US using the data from the Hispanic Community Children's Health/Study of Latino Youth. They found that the prevalence of FI was high, 46%, which was double the national average and that youth living in those food insecure households had a higher mean BMI and depression scores than their food secure peers. This was attributed to greater familial acculturative stress, greater economic stress and a weakened family support system that was more prevalent in food insecure households. It is worth noting that food insecure youth within the lowest household income was associated with greater BMI. Therefore, the authors suggested that poor diet quality and weight gain might only be associated with FI within the context of low income (29). This finding harmonizes with the work done by Oberle et al. (30) where children found to be food insecure had significantly higher BMI percentiles, although household income was not measured.

Most studies have found that the paradox is present only in women and not in men. For this reason, Taylor and colleagues conducted a study to explore the factors that may explain this gender disparity and recruited a total of 25 food insecure mother-father pairs in Connecticut, USA. Participants were interviewed individually using the United States Department of Agriculture Household Food Security Module, Center for Epidemiological Studies Depression Scale, and Coping Strategies Index. Although they did not find significant associations between BMI, FI and depression, this study offered a possible explanation for why women seem to be the only ones getting affected. They found that it was significantly more likely for mothers to sacrifice their diet quality to feed their children than fathers. The authors argued that this tendency may be attributed to social constructs were, at least in traditional gender roles, men have the place of breadwinner and providers so mothers might prioritize the needs of their husbands and children over their own needs (31).

Research has mostly focused on explaining the paradox at a household level. Farrell and colleagues reviewed the literature pertaining to low- and middle-income countries and focused on the bigger picture, that is, analyzing the issue at an individual, household, community, and country level. They proposed 5 context-mechanisms factors that could modify the association between an individual's food insecurity and obesity risk: affordability of energy dense, processed foods, quantity & diversity of food consumed, spatial temporal access to nutritious food, interpersonal distribution of food and non-dietary behavior. Nevertheless, affordability of energy dense foods was identified as the main mechanism since the authors had limited evidence to support the other mechanisms (26). Other authors have proposed that social support can also play a role since they found that food insecure women who reported lower levels of social support were more likely to be obese (28).

To the best of our knowledge, literature regarding the obesity and FI paradox in Latin American countries has not been published in recent years.

## Discussion

The present mini review had the purpose of examining the literature that has been published within the last 5 years regarding the FI– obesity paradox with the aim of improving our understanding of the topic.

As of now, most of the literature has consistently shown that there is a significant association between FI and obesity, specifically in women of high-income countries (16, 25–28, 31, 32). It is worth noting that unlike previous years, emerging studies have aimed to analyze the issue in children, adolescents, young adults, and the elderly. These studies have found the FI– obesity relationship to be true in children and adolescents (29, 30) but not in young adults (33) and the elderly (34). However, there is not enough information to draw strong conclusions in these segments of the population.

Mechanisms explaining the paradox are still lacking. Even though researchers have tried to analyze the issue using different individual and societal variables, these studies have failed to explain the mediatory mechanisms of the food insecurity–obesity relationship since the proposed mechanisms usually lack strength (25, 26) or are purely theoretical (16). This could be partly explained by the nature of their methodology; most studies used a cross-sectional model analyzing data drawn from other research that did not have the purpose of directly studying the phenomenon, for instance, data from large national health surveys.

Until now, studies have suggested that food insecure populations are more likely to have access to high-energy, processed foods (because of their affordability), thereby increasing their likelihood of becoming obese. Although being a sound argument, there is no strong evidence to support this claim since there are no studies which have measured food intake, household income and living expenses at the same time so to demonstrate that in fact a healthier diet could be more expensive. Perhaps is not that they lack the resources but the knowledge or abilities to make better food choices.

There are several research barriers and challenges regarding the paradox. For instance, there is no gold standard when it comes to measuring food insecurity, since there are instruments that range from a single question to 10 – 18 items questionnaires and there is no clear way of categorizing the severity and duration of such insecurity (acute vs. chronic) (35). In addition, when selecting study populations, factors such as physical activity, sex, age, height and eating behaviors ought to be considered for they affect energy balance. Another challenge is that in order to examine how FI could lead to obesity, there is the need for longitudinal studies.

In conclusion, although much of the literature supports the idea that FI is associated with obesity, it has only been consistent in women from high income countries, particularly from the U.S. The research focus should shift from cross-sectional models to other research designs that allow the exploration of pathways and mechanisms underlying the FI and obesity relationship, which will hopefully lead to consecutive research testing the effectiveness of different approaches and scale up such interventions into diverse contexts among those who experience obesity and the varying degrees of food insecurity.

## Author contributions

All authors listed have made a substantial, direct, and intellectual contribution to the work and approved it for publication.

## Conflict of interest

The authors declare that the research was conducted in the absence of any commercial or financial relationships that could be construed as a potential conflict of interest.

## Publisher's note

All claims expressed in this article are solely those of the authors and do not necessarily represent those of their affiliated organizations, or those of the publisher, the editors and the reviewers. Any product that may be evaluated in this article, or claim that may be made by its manufacturer, is not guaranteed or endorsed by the publisher.
